# Vestibular compensation: extended review

**DOI:** 10.3389/fneur.2026.1769641

**Published:** 2026-02-05

**Authors:** O. Nuri Özgirgin, Badr Eldin Mostafa, George M. Zaytoun, Alfarghal Mohamad, Henda Ben Hassouna Gouider, Anis Bouazzaoui, Louis Murray Hofmeyr, Nargiza Abdullayevna Karimova, Mohamed Fawzy, Sameer Qureshi

**Affiliations:** 1 Department of Otolaryngology, Head and Neck Surgery, Bayındır Hospital, Ankara, Türkiye; 2 Department of Otorhinolaryngology Head and Neck Surgery, Faculty of Medicine, Ain-Shams University, Cairo, Egypt; 3 Department of Otorhinolaryngology, Head and Neck Surgery, American University of Beirut Medical Center, Beirut, Lebanon; 4 Department of Ear, Nose, Throat, King Abdul Aziz Medical City, Jeddah, Saudi Arabia; 5 Private Practitioner, Tunis, Tunisia; 6 Private Practitioner, Rabat, Morocco; 7 Division of Otorhinolaryngology, Stellenbosch University, Stellenbosch, South Africa; 8 Department of Otorhinolaryngology, Head and Neck Diseases, Republican Specialized Scientific and Practical Medical Center of Otorhinolaryngology, Head and Neck Diseases, Tashkent, Uzbekistan; 9 Department of Vestibular Medicine and Otoneurology, Dubai Health, Dubai United Arab Emirates and MBRU/Mansoura University, Mansoura, Egypt; 10 Department of Otorhinolaryngology, Head and Neck Surgery, Hamdard University, Karachi, Pakistan

**Keywords:** vestibular compensation, vestibular disease, vestibular hypofunction, vestibulo-ocular reflex, histamine, betahistine

## Abstract

Vestibular compensation (VC) represents a remarkable aspect of neuroplasticity, showcasing the brain’s ability to adapt to disruptions in balance and spatial orientation caused by various vestibular disorders. This extended review provides a comprehensive overview of the mechanisms underlying VC, focusing on the distinct challenges posed by unilateral and bilateral vestibular disease. By examining the pathophysiological processes associated with these conditions, we gain critical insights into how the central nervous system employs adaptive strategies to restore functional balance. Additionally, this review underlines the multifaceted nature of VC, which emphasizes the necessity for personalized approaches in treatment, as not all patients will respond similarly to therapeutic interventions. Advancing our understanding of VC enriches the field of neurorehabilitation and holds significant promise for improving the quality of life for patients affected by vestibular disorders. By continuing to explore the intricate mechanisms of compensation and the factors that influence recovery, we can enhance our approaches to diagnosis, treatment, and rehabilitation. This will ultimately lead to better patient outcomes and a deeper comprehension of the brain’s remarkable adaptability in the face of vestibular challenges. The journey toward improved care for individuals with vestibular disorders is ongoing, and it is imperative that we remain committed to research, education, and innovation in this vital area of medical science.

## Introduction

Vestibular compensation (VC), a dynamic and intricate process, plays a pivotal role in restoring equilibrium and spatial orientation in patients grappling with vestibular disorders. The vestibular system serves as a crucial component in maintaining balance and coordinating eye movements to stabilize visual perception during head movements. Disruptions to this delicate system, whether due to vestibular end-organ inflammation, tumors affecting the vestibular nerve, surgery or trauma affecting the vestibular end organ, chronic progressive diseases, vestibulotoxicity, can result in debilitating symptoms such as dizziness, vertigo, and postural instability.

In response to these challenges, the brain initiates a remarkable series of adaptive mechanisms known as VC. This compensatory process involves intricate interactions between the vestibular nuclei, cerebellum, and cortical regions to recalibrate sensory inputs, suppress maladaptive reflexes, and facilitate neural plasticity. Through a combination of gaze stabilization, postural adjustments, and sensory reweighting, individuals can gradually overcome the sensory mismatch and regain functional independence during daily activities.

This narrative review aims to provide a comprehensive overview of VC, delving into the neural pathways, cellular mechanisms, clinical implications, treatments and future directions in the field of vestibular rehabilitation (VR).

## Unilateral versus bilateral vestibular disease

Patients with unilateral vestibular hypofunction (UVH) experience vertigo and/or dizziness, accompanied by neurovegetative symptoms such as nausea and vomiting. These conditions are highly disabling, leading to increased anxiety and depression (1).

Vestibular disease, seen in individuals experiencing acute UVH, includes both static manifestations evident during periods of inactivity and dynamic symptoms triggered by movement of the patients’ head or entire body (2).

According to the Barany society criteria, bilateral vestibulopathy (BVP) is a chronic vestibular syndrome with the following manifestations (3):

- Movement-induced blurred vision or oscillopsia during walking or quick head/body movements and/or worsening of unsteadiness in darkness and/or on uneven ground.
- No symptoms while sitting or lying down under static conditions.
- Bilaterally reduced or absent angular vestibulo-ocular reflex (VOR) function documented by video head impulse test (vHIT) (VOR gain <0.6), caloric test or rotatory sinusoidal chair.

Symptoms of BVP are insidious and dominated by imbalance (91.4%) and chronic dizziness (57.7%), both worsening in the dark and on uneven grounds leading to high risk of falls (42%) with significant socio-professional consequences, including the inability to work and social isolation in many cases (4, 5).

Another major symptom of BVP is oscillopsia during active head or body movements especially when walking, because the impaired VOR fails to drive the compensatory eye movement adequately so the patient will perceive the scene as swaying or bouncing with each step, this symptom worsens with abrupt movements such as riding a vehicle on a bumpy road (3, 6). Diminished dynamic visual acuity (DVA) is another symptom of BVP especially in dynamic situations. In contrast, for slow and low frequency head movements, the smooth pursuit system can stabilize fixation when a visible target is present. In rare cases oscillopsia can even occur in synchrony with the heartbeat (3).

Bilateral vestibular hypofunction (BVH) is a heterogeneous clinical condition regarding etiology and progression pattern. Four different clinical subtypes have been defined; recurrent vertigo with BVH, rapidly progressive BVH, slowly progressive BVH, and BVH with neurological deficits (7). A new study showed that most non-idiopathic BVH patients presented a clinical subtype that would be expected from its etiology (8). Most common etiologies of BVH are idiopathic (51%), toxic/metabolic (13–21%), infectious (3.8–12%), autoimmune (10%), neurodegenerative, genetic, vascular and neoplastic (7).

The Bruininks-Oseretsky Test of motor proficiency, vestibular evoked myogenic potentials (cervical, ocular and recently masseter), the video ocular counter-roll test, and functional gait assessment represent recently adopted modalities for the evaluation of BVH (8–11).

Vestibular compensation in different neuronal sites restores VOR function to some extent over time in UVH patients, while VOR gains remain markedly diminished or absent in BVH patients (12, 13).

## Compensation

After UVH, a significant imbalance in resting discharge of vestibular nuclei (VN) occurs and a series of molecular and cellular phenomena take place during early hours and days in the ipsilesional VN and related structures such as vestibulocerebellum, thalamus, vestibular cortex, hippocampus and amygdala. This leads to a functional and structural reorganization in the ipsilesional VN complex and fast rebalancing of spontaneous resting discharge on both sides with as a consequence the improvement of static symptoms. Dynamic symptoms improve in a slower fashion relying on the elaboration of new operating modes, but never fully compensate (1).

Sensory substitution takes place over the long term by means of reweighing extra-vestibular inputs such as visual cues, somatosensory inputs and neck proprioceptive drive onto ipsilesional VN. This allows substitution for earth modality in controlling posture and trunk stability (1, 14, 15).

On the other hand, the insidious symptoms of BVH makes access to early stages of compensation in humans challenging. As a result, compensation mechanisms involved in BVH can be studied better in animal models (13). After bilateral labyrinthectomy, animals are severely impaired in the first 3 days, after a week ataxia reduces significantly, however dynamic postural instability persist over time (16).

Patients with BVH can typically maintain a normal stance in light. However, their risk of falling significantly increases when standing on a moving surface (17). In situations where the surface is tilted, BVH subjects with closed eyes tend to either sway with the platform movement at lower peak tilt angular velocity or experience falls at higher peak tilt angular velocity (6).

### Static and dynamic compensation after vestibular dysfunction

Unilateral or bilateral peripheral vestibular system damage is followed by compensatory plasticity involving multiple vestibulo-ocular, vestibulo-spinal, vestibulo-collic and vestibulo-autonomic responses. These compensatory mechanisms are produced by cascades of molecular events in single cells, which take place within the context of that neuron in functional networks that produce behavioral compensation. Therefore, identification of molecular basis for a specific compensatory response (for example; disappearance of spontaneous nystagmus in the light, disappearance of static head tilt or modification of orthostatic responses) requires careful attention to the time course of behavioral compensation and the location of the effects within neural networks which normally modulate and coordinate the responses (18, 19).

In general, UVH causes spontaneous nystagmus, shifts in postural regulation, and locomotor deviation. These changes are based on the plasticity of the central vestibular system which is referred to as VC (18).

Unilateral labyrinthectomy (UL) introduces a loss of resting activity in numerous neurons of the ipsilateral medial vestibular nucleus, resulting in an imbalance in neural activity between the VN on each side of the brainstem. This imbalance induces spontaneous nystagmus. During the initial process of VC after UL, suppression of the contralateral medial vestibular nucleus by the vestibular cerebellum repairs the imbalance in VN activity. In comparison, compensation for the dynamic symptoms is less dependent on the rebalancing of electrical activity in the VN complexes (19).

It is validated that the rehabilitation of vestibular deficit patients must start as soon as possible. And for better results, rehabilitation programs should include active training to involve all sensory cues. Pharmacological treatment can also assist the VC process.

### Vestibulo-ocular reflex gain and catch-up saccades during compensation

In real life, VOR is a rather complex system of interacting elementary reflexes that utilize information from 10 sensors in two labyrinths to control 12 muscles in two eyes. At the same time, VOR is easily modified by training, which makes it a particularly tempting object for studying motor learning processes, localization of plasticity sites and elucidation of their mechanisms, which is the basis for rehabilitation of the vestibular system (20).

A study was aimed to determine whether active or passive VOR training affects how the active or passive VOR responses adapt and retain that adaptation. This study used the unilateral incremental VOR adaptation technique and exposed subjects to active or passive head impulses, while visually tracking a laser target that moves in the opposite direction to the head at a gradually increasing percentage of head velocity. A significant VOR gain on one side was observed even after 15 min of training. The short-term persistence of the increase in active and passive VOR gain was measured. This was probably the first study investigating the effect of the training context of passive and active head movements on VOR adaptation (21).

Numerous studies have shown that VOR is the only system that maintains stable vision during rapid head turns. The VOR gain (eye/head velocity) can be increased with a visual-vestibular mismatch stimulus (22).

### Compensation following destructive treatment of vestibular disorders

After destructive treatment, such as vestibular neurectomy or labyrinthectomy, the brain must rely on the remaining functioning vestibular structures as well as visual and somatosensory inputs to regain balance and stability (23). This compensation process involves reorganizing neural pathways and recalibrating sensory information to adapt to the altered vestibular signals.

The fundamental advantage of VR for compensation following destructive treatment of vestibular disorders is undisputed. Literature also supports VR for compensation, even when performed before pharmacological treatment. What is not so clear, however, is whether the addition of medical treatment improves the outcome.

### Compensation in central vestibular disorders

Central vestibular connections are continuously active to estimate the body’s orientation in regard to gravity, the direction of movement and body’s position relative to surrounding landmarks (24–26). They modulate both reflexive sensorimotor and cognitive functions including self-motion perception, bodily self-consciousness, spatial navigation, spatial learning, spatial memory, movement correction in response to changing environment, object recognition memory, numerical cognition and even economic decision making (27). On the other hand, vestibular perception may be swayed by psychological factors such as mental imagery, viewpoint changes, personality traits and social factors as well as previous experience and training (28).

Although vestibular information is bilaterally processed, there is a hemispheric dominance: it is the right hemisphere in right handers and vice versa. The ipsilateral input from the stimulated end organ is the stronger input. However, there is an integration of perception and action within a subcortical network via the corpus callosum so that there is a single “global vestibular percept” (29).

The central vestibular pathways can be affected by vertebrobasilar insufficiency, infarcts, multiple sclerosis, Parkinson’s disease, or traumatic brain injuries. Some higher vestibular hypofunctions (VH) are manifested as cognitive symptoms caused by a peripheral vestibular disorder as in Meniere’s disease, vestibular migraine, BVP and persistent postural-perceptual dizziness (30, 31).

After damage to the peripheral vestibular system, a spontaneous functional recovery or VC occurs. This is based on restoration of function, habituation, and adaptation (sensory and behavioral substitution). All involve various degrees of cortical and subcortical mechanisms. These vary with extravestibular inputs and age (32, 33). There is activation of ascending vestibular projections, recruitment of bilateral multisensory and motor-executive networks with recalibration and reorganization of brainstem, cerebellar and thalamocortical multisensory networks during the course of successful VC (34).

Central vestibular diseases impair the superior center of VOR, weakening its inhibitory effect. As a result, VOR is hyperactive and with resting nystagmus. Cerebellum plays an important role in the merging of sensory and motor signals, coordination of motor functions and cognition (35).

### Assessing central vestibular compensation: a comprehensive evaluation of vestibular dysfunction

The assessment and monitoring of CVC for static symptoms (spontaneous nystagmus and head tilt) is more straightforward compared to the dynamic component, mainly due to the intricate neuronal network involved in the latter (36).

Central vestibular compensation processes encompass a spectrum of parallel and multifaceted mechanisms. These include adaptive modifications in sensitivity and resting activity of VN and commissural networks as a reaction to signals from vestibular end organs, inhibitory responses coordinated by the cerebellum, and processes like gliosis and neurogenesis within the affected VN. Adaptation, restoration, and habituation emerge as prominent components of CVC. In these cases, CVC can be measured by a directional preponderance in caloric test, sensory organization test scores in posturography, and VOR gain and re-fixation saccades during VOR testing (36).

Expanding further into the realm of VOR gain and compensation, it becomes evident that the VOR gain’s correlation with the progression or regression of vestibular symptoms varies across clinical presentations. Temporary vestibular disorders like vestibular neuritis, exhibit an improvement in VOR gain on the lesion side. For instance, in a study, patients with unilateral vestibular neuritis demonstrated a significant increase in VOR gain on the lesion side after a 6-month follow-up. This improvement was attributed to a combination of both peripheral and central mechanisms (37).

It is important to mention the interconnected nature of compensation parameters. In patients with UVH, the non-lesioned side exhibits VOR gain, contributing to improved symmetry and CVC. In scenarios involving reduced VOR input, the cerebellum orchestrates gait adjustments through vestibular-mediated postural reflexes, mitigating gaze-induced instability (38). Studies on canal paresis recovery groups further substantiate the role of central reweighting, where the cerebellum preserves ambulation by adjusting contralesional VOR input following UVH (39).

The evolution of testing methodologies, particularly with the advent of vHIT, introduces a paradigm shift in measuring compensation. The capability to assess especially VOR reflex gain, each of the six semicircular canals separately amplifies the precision of compensation evaluation, particularly in cases of single canal pathology (40).

When dealing with transient UVH, ipsilateral VOR gain reflects CVC. In cases of permanent VH, VOR pattern in the contralateral side is most telling of CVC.

Several critical parameters are used to assess catch-up saccades (CS), including CS velocity, prevalence or percentage, amplitude, and latency of the saccade component. It was found that, even in cases where there is no improvement in VOR gain, altered CS velocity contributes to gaze stabilization and vestibular symptom improvement. A study on patients undergoing vestibular schwannoma resection revealed a significant reduction in overt saccade velocity during an ipsilesional passive head turn after 5 weeks of VR training (41). Additionally, a gradual reduction in covert and overt saccade velocities over a 6 month follow-up period was observed (36, 37). Apart from velocity, there was a gradual reduction in CS prevalence and amplitude. The prevalence of CS and modification of CS amplitude for both covert and overt saccades are crucial in compensating for retinal slip and expediting image stabilization. A higher prevalence and amplitude of overt CS was found in patients requiring VR training compared to well-compensated individuals. It also correlated with a higher degree of VH translated by higher scores on Dizziness Handicap Inventory (DHI) and lower dynamic visual acuity scores (42, 43).

Introducing the Functional Head Impulse Test (FHIT) into the compensation assessment toolkit represents a notable advancement. Unlike traditional testing methods, FHIT incorporates both passive and active head movements, mimicking real-life scenarios (44). The evaluation of vestibular functions concerning varying angular head acceleration during visual tasks adds a layer of complexity to compensation assessment. The findings from FHIT underscore the role of cortical involvement in compensation, especially during active head impulses. This test is unique in that it measures CVC by evaluating visual acuity rather than ocular movement (45).

Computerized dynamic posturography (CDP) is a specialized assessment tool, also employed in the evaluation of CVC, especially during the rehabilitation process. CDP utilizes a force platform to analyze an individual’s postural responses under diverse sensory conditions, challenging the vestibular system and assessing the effectiveness of compensatory mechanisms. This technology quantifies an individual’s ability to maintain balance in real-time, providing objective measurements of postural stability (46).

Nowadays, with the introduction of virtual reality, replication of real-world scenarios during testing and VR has improved the use of CDP. Virtual reality is able to manipulate tasks and environments to evaluate functional postural behavior. It is also a practical tool for the testing of postural disorders as a result of sensory mismatch. In the appropriate setting, it delivers an integral discordance between visual and vestibular senses and can improve the efficacy of CDP in evaluation and treatment (47).

## Treatment

Vestibular rehabilitation is the primary treatment of manifestations and functional limitations arising from vestibular deficits. It has demonstrated efficacy in improving dizziness, enhancing postural stability, reducing the risk of falls, and improving visual acuity during head movements in individuals with VH (48).

A revised Cochrane Review from 2015 reports the moderate to strong evidence supporting VR in the treatment of UVH. This comprehensive analysis concludes that such rehabilitation efforts exhibit a moderate to strong efficacy in symptom reduction and functional improvement (49). Additionally, a recent systematic review has affirmed strong evidence supporting the effectiveness of vestibular exercises for individuals with BVH, particularly in the enhancement of gaze and postural stability (50).

Both UVH and BVH patients show improvement in dynamic gait stability and dynamic visual acuity (51). Video head impulse test offers an interesting tool to evaluate the extent of central compensation by measuring VOR gain improvement in temporary UVH, and in permanent UVH and BVH CS percentage, velocity, amplitude, latency and Perez and Rey score (36, 52). However, some patients with severe bilateral hypofunction have poor compensation and therapeutic options are limited. This group of patients constitutes the group of interest for clinical research and rehabilitation with prosthetic devices (48, 50).

Vestibulo-ocular reflexes stabilize the gaze above 2 Hz motion and enable clear vision (53). For clear vision, eye movements should compensate for head movements. Vestibular nerves discharge in synchrony at a rate of about 90 action potentials per second in the lack of motion. This can either be decreased or increased during the rotation of the head in one direction or the other. Angular and translational VOR induce eye motion to compensate for head rotation and translation, and to stabilize gaze direction. The modulation of these reflexes could be used to restore impaired vestibular function which makes the basics of VR (54).

Vestibular rehabilitation aims adaptation and motor learning. Gaze stabilization, habituation, balance training and application to increase endurance are the fundamental steps of VR. It improves postural stability, decreases disequilibrium and improves VOR.

Gaze stabilization targets VOR adaptation and substitution, visual clarity during head motion, support to vestibulo-ocular gain combination of gaze and somatosensorial stabilization, and combination of gaze and walking. To enable stabilization, it is important to keep one’s eyes to stay on a target during head movement. This will be possible by keeping the eye fixed on the target in motion. Creation of retinal slip is the key activity. Retinal slip signal is carried by Purkinje cells that code the information to trigger plasticity. To keep the eye still in space during head movement, eye movement velocity should be exactly opposite to head motion. Retinal image motion must be less than 2° per second for maintaining normal vision.

Decreasing visual clearance disturbances, desensitization with repetitive exercises, VOR, balance and optokinetic stimulation exercises and optokinetic wide field stimulation serve for habituation.

Balance training to reduce postural asymmetry and perturbations is essential for VR. Stabilization, weight transfer to increase limits of stability and to reduce sway, weight holding, and postural control are essentials for postural biofeedback.

Conventional VR has always been advantaged as being interactively supervised (55). Besides the supervised rehabilitation techniques, introduction of dynamic posturography additionally reveals gaze stabilization and habituation techniques over and above serving as a valuable device to monitor the progress of VR (56).

Virtual reality is a powerful tool to create natural surroundings and controlled circumstances. It serves for application of various levels of difficulties by creating perceptual confusion and supports VOR and vestibulospinal reflex gains. Repeated motion of objects on the retina helps vestibular adaptation by visual stimulation. No major side effects of virtual reality applications have been reported (57).

The efficacy of VR depends on the timing of initiation, motivation of the patient and affected site. Patients need to continue a controlled VR training program and to undergo regular quantitative assessments of postural control. These assessments are necessary to evaluate their level of instability and their response to rehabilitative treatment (58). Failure of rehabilitation may be due to anxiety, depression, decompensation, or a maladapted rehabilitation program as well as degenerative diseases of the central nervous system (CNS) and neuromuscular disorders (59, 60).

For an optimal functional recovery, it is essential to portray the sensorimotor and cognitive profile of each patient and define the external factors that influence recovery. A customized VR program, depending on the assessment of the patient may include:

1. VOR training with eye-head coordination exercises.
2. Training of conjugated eye movements with smooth pursuit and saccadic movements.
3. Optokinetic training.
4. Positional habituation exercises.
5. Postural control training.
6. VOR suppression and VOR memory.
7. Anti-saccade and memory-guided saccades.
8. Stretching and strengthening exercises.
9. Graduated movements with yoga and tai chi.

Integrating novel technologies—like immersive virtual reality, internet/mobile phone applications, and sensory and vibrotactile enhanced biofeedback, vestibular stochastic resonance training or cerebellar intermittent theta burst stimulation—into VR can improve dizziness, balance, gait, impact of fatigue and quality of life (QoL) and provide the best and personalized VR training for patients with central VH (35, 58, 61, 62).

Vestibular implants have emerged as an important therapeutic option for the management of BVH refractory to conservative treatments, including VR. Unlike cochlear implants, vestibular prostheses rely on head-motion sensing to enable selective stimulation of the three divisions of the vestibular nerve. Head movements are captured by motion sensors and processed externally into patterned electrical signals, which are wirelessly transmitted to an implanted stimulator equipped with vestibular electrodes. Electrical stimulation delivered through these electrodes evokes action potentials in the vestibular nerve, facilitating restoration of vestibular function.

Implementation of a vestibular prosthesis can result in recovery of VOR and may also enhance perceptual and postural control in patients with BVH (63, 64). Over recent decades, multiple research centers have pursued the development of vestibular implants. Investigators at the University of Washington developed a vestibular neurostimulator derived from a commercially available cochlear implant that was modified to stimulate semicircular canal afferents (65). The Maastricht-Geneva group subsequently introduced the vestibulocochlear implant, which employed separate wire electrodes to electrically stimulate the ampullary nerves (66). A multichannel vestibular implant was developed at Johns Hopkins University, incorporating integrated gyroscopes and accelerometers capable of detecting and encoding three-dimensional angular rotations and linear accelerations (67). The otolith implant represents the most recent vestibular implant modality and was developed to provide direct otolithic vestibular stimulation. Electrical stimulation of the vestibular nerve using an otolith-based approach has demonstrated a substantial functional impact on balance, performance of daily activities such as walking, and fall prevention (68). These studies are promising for QoL improvement, with a theoretical potential to improve CVC; however, these outcomes have not yet been directly investigated in published studies (69, 70).

### Neuropharmacological approach

The aim of pharmacological intervention should be to suppress inappropriate central responses and enhance repair processes. For example, gamma-aminobutyric acid (GABA) agonists like benzodiazepines and baclofen can suppress unwanted central processes whereas drugs that increase compensation (drugs that increase dopamine or norepinephrine) and histamine agonists may be useful. Some drugs like 4-aminopyridine, n-acetyl leucine, nicergoline and gingko biloba have some potential in the management of central vestibular disorders and may enhance recovery and/or compensation (71–74).

### Histaminergic system and the vestibule

Histamine is a biogenic amine and signaling molecule that is widely distributed throughout the body, including the inner ear. It plays a diverse and essential role in various physiological processes throughout the body, including neurotransmission, gastric acid secretion, inflammation, vasodilation and smooth muscle contraction. Histamine is not only a neurotransmitter, but also a mediator of inflammation that plays various roles in the body, including regulating allergic reactions and triggering pruritus. Its actions are mediated through interactions with specific histamine receptor subtypes, making it a key target for pharmacological interventions in the treatment of allergic and inflammatory conditions (75, 76).

In the CNS, histamine acts as a neurotransmitter involved in regulating wakefulness, arousal, and attention. It is synthesized primarily by neurons in the hypothalamus and projects widely throughout the brain (77). Histamine signaling is mediated by four distinct receptor subtypes (H1, H2, H3, and H4), each with unique distribution and functions (76). Histamine is known to affect vestibular function through several mechanisms including neurotransmission, blood flow regulation and inflammation. Activation of histamine receptors can modulate the release of neurotransmitters such as glutamate and GABA, which are essential for signal transmission between vestibular hair cells, vestibular ganglion cells, and central vestibular pathways. Alterations in histamine levels could reflect underlying inflammatory processes, neurotransmitter imbalances, or vascular changes within the vestibule (75).

As histamine receptors are expressed throughout the vestibular system, changes in receptor expression patterns may be indicative of specific vestibular pathologies. Immunohistochemical analysis of vestibular tissue samples could reveal alterations in histamine receptor distribution in patients with vestibular disorders. For instance, upregulation of histamine H3 receptors in the VN may be associated with VC following peripheral vestibular injury, while downregulation of H1 receptors in vestibular hair cells may contribute to VH. Emerging evidence suggests that H4 receptors may be involved in vestibular inflammation and immune responses. Inflammatory processes within the vestibule can disrupt the delicate balance of fluid and ion homeostasis, leading to VH. By regulating the release of pro-inflammatory cytokines and chemokines in response to vestibular injury or insult, H4 receptors may contribute to these inflammatory processes (75).

Targeting histamine receptors within the vestibule holds promise for the management of various vestibular disorders. By modulating neurotransmission, inflammation, and immune responses, histamine receptor-based therapies offer diverse therapeutic strategies aimed at alleviating symptoms and improving QoL for individuals with VH.

H1 receptor antagonists, such as meclizine and dimenhydrinate, are commonly used as antiemetic agents to alleviate symptoms of motion sickness and vertigo associated with vestibular disorders (78).

H3 receptor antagonists have been investigated for their potential in enhancing VC processes. By increasing histamine release, these agents may promote neuronal plasticity and facilitate the central adaptation to VH. Also, H3 receptor antagonists block presynaptic histamine H3 autoreceptors, leading to increased histamine release and enhanced neurotransmission (79).

Betahistine (*N* alpha-methyl-2-pyridylethylamine), is a structural analog of histamine. It was initially introduced to be used as a therapy for vascular and vasomotor disorders such as cluster headaches and vascular dementia. It is an H3 receptor antagonist with weak H1 receptor agonist activity, and widely used to alleviate symptoms of vertigo, dizziness, and nausea in vestibular disorders like Meniere’s disease and vestibular migraine (80).

Betahistine has been reported to increase cochlear blood flow via presynaptic H3 heteroreceptors and autonomic α_2_ receptors and by vasodilation of anterior inferior cerebellar artery by 17–20% (81).

An additional mechanism of action is betahistine’s effect on the histaminergic system, leading to increased histamine synthesis and release in the tuberomammillary nuclei of the posterior hypothalamus, which explains the neuromodulatory effects of betahistine on peripheral and central vestibular neuronal activities (82).

Even though a Cochrane review concluded that there was insufficient evidence regarding the effect of betahistine on objective tests of vestibular function, and there was no available data on its impact on overall QoL or fall risk, it has to be kept in mind that this Cochrane review was based on the effect of betahistine only on vertigo and dizziness (83). The pathophysiological patterns affecting the active disease is different in the compensation period. A preliminary study demonstrated that patients receiving early VR in combination with betahistine therapy experienced the fastest recovery. Moreover, following head trauma, compared with betahistine monotherapy, early VR was associated with a statistically significant acceleration of balance recovery (84). With all the data we have in hand, the effect of betahistine as a monotherapy in improving VC needs further study and early VR in combination with betahistine therapy may be associated with improved recovery (83–85).

## Discussion

Vestibular compensation represents a remarkable aspect of neuroplasticity, showcasing the brain’s ability to adapt to disruptions in balance and spatial orientation caused by various vestibular disorders. This extended review has provided a comprehensive overview of the mechanisms underlying VC, focusing on the distinct challenges posed by unilateral and bilateral vestibular disease. By examining the pathophysiological processes associated with these conditions, we gain critical insights into how the CNS employs adaptive strategies to restore functional balance.

Our exploration of UVH has illuminated the brain’s intricate recalibration processes following the loss of input from one vestibular system. Central to this adaptation is the activation of compensatory pathways, particularly involving structures such as the cerebellum, brainstem, and VN, which collectively facilitate the restoration of equilibrium and the minimization of symptoms. The brain’s capacity to harness various sensory inputs and integrate them effectively underscores the complexity of vestibular function and highlights the resilience of neural networks in response to injury.

Conversely, in the context of bilateral vestibular disease, the absence of input from both vestibular systems introduces a unique set of challenges. Patients with bilateral deficits often experience profound and debilitating symptoms, including significant balance impairments and spatial disorientation. The compensatory mechanisms in these cases are more intricate and may involve greater reliance on visual and somatosensory systems. Understanding these differences is essential for developing targeted rehabilitation strategies that address the specific needs of patients based on the nature and severity of their vestibular dysfunction.

Additionally, this review has underlined the multifaceted nature of VC, which emphasizes the necessity for personalized approaches in treatment, as not all patients will respond similarly to therapeutic interventions. Clinicians must consider these factors when designing rehabilitation programs to optimize recovery and enhance functional outcomes. Vestibular rehabilitation therapies, which encompass a variety of interventions including balance training, habituation exercises and gaze stabilization techniques have been shown to be effective in enhancing compensation and improving the QoL for many patients. These therapies not only address physical symptoms but also promote psychological resilience by empowering patients to reclaim their independence and confidence. Pharmacological interventions also play a crucial role in symptom management, addressing issues such as dizziness, nausea, and anxiety.

As we look toward the future, further research is warranted to investigate the underlying biological processes that govern VC. Longitudinal studies will be essential in understanding the long-term outcomes of various treatment strategies, particularly in relation to early intervention and its impact on recovery trajectories. Moreover, the incorporation of novel technologies, such as virtual reality, telehealth, and robotic assistance, may revolutionize VR by providing more engaging, immersive, and effective therapeutic options. Recent advances in artificial intelligence may also provide new approach to VR. These innovations could enhance patient adherence and motivation, ultimately leading to better clinical outcomes.

Furthermore, interdisciplinary collaboration holds great promise for advancing our knowledge and treatment of vestibular disorders. By fostering partnerships among audiologists, neurologists, physical therapists, and psychologists, we can develop more holistic approaches that address the diverse needs of patients. In addition, integrating patient-reported outcomes and experiences into research and clinical practice will ensure that treatments are not only effective but also aligned with the goals and preferences of those affected by vestibular disorders.

In conclusion, advancing our understanding of VC enriches the field of neurorehabilitation and holds significant promise for improving the QoL for patients affected by vestibular disorders. By continuing to explore the intricate mechanisms of compensation and the factors that influence recovery, we can enhance our approaches to diagnosis, treatment, and rehabilitation. This will ultimately lead to better patient outcomes and a deeper comprehension of the brain’s remarkable adaptability in the face of vestibular challenges. The journey toward improved care for individuals with vestibular disorders is ongoing, and it is imperative that we remain committed to research, education, and innovation in this vital area of medical science.
